# Draft Genomes of 92 *Vibrio* Isolates from Warm-Water Shrimps Imported into Canada between 2009 and 2019

**DOI:** 10.1128/mra.00750-22

**Published:** 2022-11-14

**Authors:** Januana S. Teixeira, Forest Dussault, Emily Hoover, Julie A. Shay, Kelly Weedmark, Swapan K. Banerjee

**Affiliations:** a Bureau of Microbial Hazards, Health Canada, Ottawa, Ontario, Canada; b Bureau of Food Surveillance and Science Integration, Health Canada, Ottawa, Ontario, Canada; Indiana University, Bloomington

## Abstract

*Vibrio* spp. were isolated from raw shrimps imported into Canada (2009 to 2019). A total of 92 isolates with various multidrug resistance profiles were sequenced, including 59 V. parahaemolyticus, 12 V. diabolicus, 10 V. cholerae, 7 V. alginolyticus, 1 V. campbellii, 1 V. harveyi, 1 V. owensii, and 1 V. vulnificus isolate.

## ANNOUNCEMENT

*Vibrio* spp. are Gram-negative bacterial pathogens ubiquitous in aquatic and marine environments (1). They can cause severe gastrointestinal infection in humans from ingestion of water or raw/partially cooked seafood contaminated with pathogenic *Vibrio* spp. (2). The majority of human illnesses associated with *Vibrio* spp. are caused by V. cholerae, V. parahaemolyticus, V. vulnificus, and V. alginolyticus (1). Given a propensity for horizontal gene transfer among *Vibrio* spp. and the presence of antimicrobial-resistant (AMR) bacteria in marine ecosystems (2–4), *Vibrio* spp. have the potential to acquire resistance to antibiotic treatment (4). This study sequenced and identified 92 *Vibrio* isolates present in raw shrimp imported to Canada that were selected based on their antibiotic resistance profiles.

Single colonies from imported raw shrimp samples (*n* = 54, fresh or frozen) were isolated as in reference 5. Briefly, shrimps (50 g/sample) were homogenized in a blender with 450 mL alkaline peptone water (1% Bacto peptone, 2% NaCl, pH 8.5) and equilibrated for 1 h at 21°C before overnight enrichment at 35°C. Selective agars (thiosulfate citrate bile salt sucrose [TCBS], CHROMagar, and mCC10 or CC400) were used for both pre-enrichment (100 μL) and postenrichment (10 μL) plating. After 35°C overnight incubation, presumptive *Vibrio* colonies were grown at 35°C overnight on tryptic soy agar with 2% NaCl (TSA-2N) (Difco BD, Franklin Lakes, NJ, USA) for molecular and biochemical *Vibrio* confirmation (5) and −80°C stocks (10 μL loopful/Microbank vial) (VWR, Mississauga, Ontario, Canada). The AMR phenotype to 23 antibiotics (Oxoid Ltd., Basingstoke, UK) was determined for isolates using the Kirby-Bauer disk diffusion method and published guidelines (6–8); a subset of 92 isolates was selected for sequencing (Table 1).

**TABLE 1:** Genome assembly metrics for 92 *vibrio* species isolates from raw shrimp imported to Canada (NCBI BioProject PRJNA843723)

Isolate	yr	Country of origin	Taxonomic ID (MASH)	AMR profile^*a*^	Genome size (bp)	GC (%)	Coverage (×)	No. of contigs	No. of reads	*N*_50_ (bp)	No. of genes	BioSample accession no.	Assembly accession no.	WGS GenBank accession no.	SRA accession no.
ISF-4-2	2009	Thailand	V. parahaemolyticus	AMP (KF, SXT, E)	5,031,302	45.4	57	74	1,153,170	155,980	4,701	SAMN28767292	GCA_024746775	JANFVC000000000	SRR19513301
ISF-8-6	2009	China	V. parahaemolyticus	AMP (KF, CIP, OX)	4,917,741	45.5	105	38	1,873,986	353,627	4,548	SAMN28767293	GCA_024746735	JANFVB000000000	SRR19513300
ISF-10-3	2009	Vietnam	V. alginolyticus	AMP, PIP, KF	5,126,989	44.6	42	90	950,138	148,213	4,711	SAMN28767294	GCA_024746755	JANFVA000000000	SRR19513289
ISF-15-1	2009	Mexico	V. diabolicus	AMP, PIP, OX	5,159,569	44.9	82	44	1,635,904	214,849	4,795	SAMN28767295	GCA_024746715	JANFUZ000000000	SRR19513278
ISF-15-4	2009	Mexico	V. parahaemolyticus	AMP, PIP, KF, OX	5,145,313	45.2	107	62	1,997,334	193,197	4,732	SAMN28767296	GCA_024746695	JANFUY000000000	SRR19513267
ISF-24-5	2009	Ecuador	V. parahaemolyticus	AMP, PIP, KF	4,951,092	45.4	79	51	1,463,684	315,201	4,564	SAMN28767297	GCA_024746655	JANFUX000000000	SRR19513256
ISF-29-3	2009	Ecuador	V. parahaemolyticus	AMP, SF, TE, OT	5,320,303	45	28	140	713,474	108,977	4,951	SAMN28767298	GCA_024746635	JANFUW000000000	SRR19513225
ISF-29-4	2009	Ecuador	V. diabolicus	AMP, KF, OX	5,060,319	44.8	116	57	2,140,656	199,367	4,728	SAMN28767299	GCA_024746645	JANFUV000000000	SRR19513214
ISF-36-1	2010	Peru	V. diabolicus	AMP, PIP, OX	5,017,756	44.8	47	55	1,029,532	294,177	4,633	SAMN28767300	GCA_024746585	JANFUU000000000	SRR19513247
ISF-38-2	2010	Thailand	V. alginolyticus	AMP, PIP, KF	5,204,850	44.5	25	150	512,020	78,737	4,777	SAMN28767301	GCA_024746615	JANFUT000000000	SRR19513236
ISF-49-4	2011	India	V. parahaemolyticus	AMP, KF, OX	5,085,405	45.3	63	84	128,0358	202,791	4,759	SAMN28767302	GCA_024746555	JANFUS000000000	SRR19513299
ISF-49-9	2011	India	V. parahaemolyticus	AMP, KF, SF, OX	5,011,927	45.4	96	29	2,029,078	461,187	4,598	SAMN28767303	GCA_024746575	JANFUR000000000	SRR19513298
ISF-49-12	2011	India	V. diabolicus	AMP, PIP, SF, OX	5,096,250	44.8	35	98	700,098	208,287	4781	SAMN28767304	GCA_024746535	JANFUQ000000000	SRR19513297
ISF-50-4	2011	Philippines	V. parahaemolyticus	AMP, PIP, KF	5,007,265	45.4	80	34	1598,182	404,666	4,673	SAMN28767305	GCA_024746515	JANFUP000000000	SRR19513296
ISF-50-6	2011	Philippines	V. parahaemolyticus	AMP, PIP, KF	4,967,225	45.4	51	46	919,352	259,902	4,622	SAMN28767306	GCA_024746495	JANFUO000000000	SRR19513295
ISF-50-9	2011	Philippines	V. parahaemolyticus	AMP, PIP, KF, SF	4,972,944	45.4	54	38	1,102,162	258,794	4,607	SAMN28767307	GCA_024746475	JANFUN000000000	SRR19513294
ISF-56-4	2011	Thailand	V. parahaemolyticus	AMP, SXT, SF, TE, OT	5,061,529	45.3	47	71	949,142	201,099	4,727	SAMN28767308	GCA_024746455	JANFUM000000000	SRR19513293
ISF-56-5	2011	Thailand	V. parahaemolyticus	AMP, SXT, SF, OT (KF, E, ENO, TE)	5,075,302	45.3	38	79	756,372	200,930	4,765	SAMN28767309	GCA_024746415	JANFUL000000000	SRR19513292
ISF-56-8	2011	Thailand	V. parahaemolyticus	SXT, KF, SF, TE, OT (E)	5,059,887	45.4	56	110	1,087,546	124,670	4,729	SAMN28767310	GCA_024746425	JANFUK000000000	SRR19513291
ISF-56-10	2011	Thailand	V. parahaemolyticus	AMP, SXT, SF, TE, OT (KF, E, C)	5,062,302	45.4	101	50	1,841,494	377,044	4,721	SAMN28767311	GCA_024746355	JANFUJ000000000	SRR19513290
ISF-57-5	2011	Vietnam	V. diabolicus	AMP, PIP, OX	5,083,437	44.9	89	50	1,710,478	210,412	4,722	SAMN28767312	GCA_024746195	JANFUI000000000	SRR19513288
ISF-61-5	2011	USA	V. alginolyticus	AMP, PIP, KF	5,291,624	44.6	49	130	967,808	111,346	4,922	SAMN28767313	GCA_024746395	JANFUH000000000	SRR19513287
ISF-63-5	2011	Vietnam	V. parahaemolyticus	AMP, PIP, KF	4,991,923	45.4	55	65	1,048,406	191,317	4,648	SAMN28767314	GCA_024746375	JANFUG000000000	SRR19513286
ISF-64-3	2011	Thailand	V. parahaemolyticus	AMP, PIP, KF	5,121,081	45.4	113	40	2,085,744	359,986	4,798	SAMN28767315	GCA_024746335	JANFUF000000000	SRR19513285
ISF-69-8	2011	Mexico	V. owensii	AMP, SXT, SF, TE, OT (PIP, E)	6,030,242	45.4	98	67	2,247,260	252,192	5,448	SAMN28767316	GCA_024746155	JANFUE000000000	SRR19513284
ISF-74-1	2012	India	V. alginolyticus	AMP, PIP, KF	5,157,601	44.7	109	80	2,006,056	169,653	4,694	SAMN28767317	GCA_024746315	JANFUD000000000	SRR19513283
ISF-77-1	2012	Indonesia	V. parahaemolyticus	AMP, PIP, KF	5,008,845	45.3	95	51	1,756,968	404,200	4,590	SAMN28767318	GCA_024746295	JANFUC000000000	SRR19513282
ISF-77-7	2012	Indonesia	V. cholerae	AMP (PIP)	4,007,917	47.6	57	96	799,370	194,054	3,722	SAMN28767319	GCA_024746215	JANFUB000000000	SRR19513281
ISF-82-2	2012	Indonesia	V. diabolicus	AMP, PIP, KF	5,005,844	44.9	63	57	1,183,816	207,104	4,590	SAMN28767320	GCA_024746275	JANFUA000000000	SRR19513280
ISF-85-1	2012	India	V. diabolicus	AMP, PIP, KF	5,097,833	44.9	83	48	1,605,808	317,856	4,708	SAMN28767321	GCA_024746235	JANFTZ000000000	SRR19513279
ISF-88-1	2012	Thailand	V. parahaemolyticus	AMP (KF, E)	5,254,276	45.2	110	81	2,321,878	216,128	4,957	SAMN28767322	GCA_024746175	JANFTY000000000	SRR19513277
ISF-93-4	2012	Vietnam	V. diabolicus	AMP, PIP, KF	5,027,740	44.9	30	93	618,270	155,061	4,643	SAMN28767323	GCA_024746245	JANFTX000000000	SRR19513276
ISF-94-3	2012	Bangladesh	V. parahaemolyticus	AMP, PIP, KF	5,036,542	45.3	224	36	4,255,404	526,057	4,677	SAMN28767324	GCA_024746135	JANFTW000000000	SRR19513275
ISF-94-4	2012	Bangladesh	V. alginolyticus	AMP, PIP, KF	5,244,137	44.5	34	101	748,274	125,444	4,832	SAMN28767325	GCA_024746095	JANFTV000000000	SRR19513274
ISF-99-2	2012	Indonesia	V. parahaemolyticus	AMP, PIP, KF	5,351,538	45.3	26	208	541,802	78,827	5,114	SAMN28767326	GCA_024746105	JANFTU000000000	SRR19513273
ISF-104-5	2012	Thailand	V. parahaemolyticus	AMP, PIP, KF	4,996,778	45.4	66	34	1,329,562	378,528	4,564	SAMN28767327	GCA_024746075	JANFTT000000000	SRR19513272
ISF-113-8	2013	Mexico	V. diabolicus	AMP (KF, E)	5,162,038	44.6	56	69	1,097,316	168,689	4,778	SAMN28767328	GCA_024746045	JANFTS000000000	SRR19513271
ISF-113-9	2013	Mexico	V. diabolicus	AMP (KF, SF, SXT, E)	5,168,988	44.6	95	38	2,141,496	397,363	4,784	SAMN28767329	GCA_024746035	JANFTR000000000	SRR19513270
ISF-128-1	2013	India	V. campbellii	AMP, PIP, OX	5,963,364	45.3	57	102	1,221,224	145,273	5,551	SAMN28767330	GCA_024746015	JANFTQ000000000	SRR19513269
ISF-128-2	2013	India	V. parahaemolyticus	AMP (OX)	5,173,068	45.2	112	34	2,033,516	553,726	4,858	SAMN28767331	GCA_024745995	JANFTP000000000	SRR19513268
ISF-131-2	2013	India	V. parahaemolyticus	AMP, TE, SF, OT (KF)	5,168,805	45.2	82	52	1,499,022	268,606	4,856	SAMN28767332	GCA_024745975	JANFTO000000000	SRR19513266
ISF-136-11	2013	Ecuador	V. parahaemolyticus	AMP, TE, OT	5,289,134	45	42	55	903,106	234,466	4,893	SAMN28767333	GCA_024745955	JANFTN000000000	SRR19513265
ISF-180-2	2014	India	V. cholerae	None	4,019,634	47.6	183	77	3,157,838	278,041	3,749	SAMN28767334	GCA_024745935	JANFTM000000000	SRR19513264
ISF-184-12	2014	India	V. parahaemolyticus	AMP, PIP, KF	5,528,471	44.9	62	98	1,333,060	190,987	5,185	SAMN28767335	GCA_024745915	JANFTL000000000	SRR19513263
ISF-184-13	2014	India	V. parahaemolyticus	AMP, KF (CTX, CEF, E)	5512,355	45.0	56	201	1,430,228	62,934	5,180	SAMN28767336	GCA_024745895	JANFTK000000000	SRR19513262
ISF-188-12	2014	No data	V. parahaemolyticus	AMP, PIP, KF, S, SXT, SF, OT	5,231,654	45.2	98	92	1,827,276	163,660	4,923	SAMN28767337	GCA_024745875	JANFTJ000000000	SRR19513261
ISF-189-3	2015	Mexico	V. parahaemolyticus	AMP, KF, PB, E, TE, OT	5,158,166	45.2	36	74	805,380	185,319	4,773	SAMN28767338	GCA_024745835	JANFTI000000000	SRR19513260
ISF-189-4	2015	Mexico	V. alginolyticus	AMP, PIP, KF	5,257,285	44.6	60	116	1,188,410	101,010	4,829	SAMN28767339	GCA_024745855	JANFTH000000000	SRR19513259
ISF-189-5	2015	Mexico	V. alginolyticus	AMP, PIP, KF	5,046,083	44.6	46	62	1,078,388	213,662	4,667	SAMN28767340	GCA_024745815	JANFTG000000000	SRR19513258
ISF-199-1	2015	Vietnam	V. parahaemolyticus	AMP, KF, SF, TE, OT (CTX, CEF, E, ENO, OX)	5,330,197	45.3	39	78	881,938	269,650	5,001	SAMN28767341	GCA_024745795	JANFTF000000000	SRR19513257
ISF-199-2	2015	Vietnam	V. parahaemolyticus	AMP, SXT, SF, CIP, OX, OT (KF, S, E, ENO, NOR)	5,062,936	45.4	57	98	1,039,018	126,520	4,655	SAMN28767342	GCA_024745755	JANFTE000000000	SRR19513255
ISF-199-3	2015	Vietnam	V. parahaemolyticus	AMP, SF, TE, OT	5,335,527	45	76	86	1,477,046	528,927	4,966	SAMN28767343	GCA_024745775	JANFTD000000000	SRR19513254
ISF-199-4	2015	Vietnam	V. parahaemolyticus	AMP, KF, SF, TE, OT (CTX, CEF, E, ENO, OX)	5,333,116	45.3	30	146	670,782	103,872	5,025	SAMN28767344	GCA_024745735	JANFTC000000000	SRR19513233
ISF-199-5	2015	Vietnam	V. parahaemolyticus	AMP, SXT, SF, CIP, ENO, NOR, OX, TE, OT (KF)	5,038,673	45.5	30	238	578,680	54,328	4,655	SAMN28767345	GCA_024745715	JANFTB000000000	SRR19513232
ISF-199-6	2015	Vietnam	V. vulnificus	CIP, ENO, NOR, OX, TE, OT	4,942,439	46.8	27	161	497,672	80,843	4,537	SAMN28767346	GCA_024745695	JANFTA000000000	SRR19513231
ISF-199-7	2015	Vietnam	V. parahaemolyticus	AMP, SF, TE, OT (KF, CTX, CEF, E, CIP, ENO, OX)	5,340,468	45.3	32	101	677,388	146,316	5,020	SAMN28767347	GCA_024745655	JANFSZ000000000	SRR19513230
ISF-200-6	2015	Vietnam	V. harveyi	AMP, SXT, SF, OX, TE, OT (PIP, S, E, CIP, ENO)	5,819,105	45.0	93	45	1926336	284289	5,375	SAMN28767348	GCA_024745675	JANFSY000000000	SRR19513229
ISF-200-8	2015	Vietnam	V. parahaemolyticus	AMP, KF, SXT, SF, TE, OT (CTX, E)	5,164,514	45.3	123	58	2,523,256	220,941	4,744	SAMN28767349	GCA_024745635	JANFSX000000000	SRR19513228
ISF-203-4	2015	India	V. parahaemolyticus	AMP, KF, OX	5,284,022	45.2	35	79	864,710	258,208	4,867	SAMN28767350	GCA_024745615	JANFSW000000000	SRR19513227
ISF-203-5	2015	India	V. parahaemolyticus	AMP, KF, OX	5,286,444	45.2	83	54	1,946,386	458,527	4,868	SAMN28767351	GCA_024745535	JANFSV000000000	SRR19513226
ISF-208-1	2016	Thailand	V. cholerae	K, OX (S, E)	4,470,139	47.3	73	86	1,207,970	152,725	4,285	SAMN28767352	GCA_024745555	JANFSU000000000	SRR19513224
ISF-208-2	2016	Thailand	V. cholerae	AMP, PIP, KF, CTX, CEF, K, OX (S, SF, E)	4,441,697	47.3	68	88	1,100,812	163,543	4,237	SAMN28767353	GCA_024745565	JANFST000000000	SRR19513223
ISF-208-5	2016	Thailand	V. cholerae	AMP, PIP, KF, CTX, CEF, K, E, OX (S, ENO)	4,477,917	47.4	106	80	1,739,866	152,725	4,308	SAMN28767354	GCA_024745585	JANFSS000000000	SRR19513222
ISF-208-8	2016	Thailand	V. cholerae	AMP, PIP, KF, CTX, CEF, K, S, OX (SF, E, CIP)	4,472,093	47.4	114	80	1,874,806	214,953	4,286	SAMN28767355	GCA_024745515	JANFSR000000000	SRR19513221
ISF-209-3	2016	Vietnam	V. parahaemolyticus	AMP, PIP, KF, SF	5,198,526	45.2	53	52	1,152,550	282,081	4,820	SAMN28767356	GCA_024745495	JANFSQ000000000	SRR19513220
ISF-209-5	2016	Vietnam	V. parahaemolyticus	AMP, PIP, KF, SF	5,210,137	45.2	46	59	916,300	228,194	4,834	SAMN28767357	GCA_024745475	JANFSP000000000	SRR19513219
ISF-209-6	2016	Vietnam	V. diabolicus	AMP, SF, TE, OT (SXT, E)	5,062,782	44.7	155	44	2,856,634	269,099	4,652	SAMN28767358	GCA_024745435	JANFSO000000000	SRR19513218
ISF-210-7	2016	Vietnam	V. parahaemolyticus	AMP, S, OX (K, E, CIP, ENO)	5,134,186	45.3	41	80	805,448	182,090	4,712	SAMN28767359	GCA_024745445	JANFSN000000000	SRR19513217
ISF-210-8	2016	Vietnam	V. parahaemolyticus	AMP, KF, CIP, OX (ENO)	5,147,064	45.3	109	35	2,090,122	447,148	4,723	SAMN28767360	GCA_024745415	JANFSM000000000	SRR19513216
ISF-211-7	2016	Thailand	V. parahaemolyticus	AMP, PIP, KF, CTX, CEF (SXT, E, ENO)	5,230,424	45.3	67	133	1,251,372	89,406	4,839	SAMN28767361	GCA_024745375	JANFSL000000000	SRR19513215
ISF-214-8	2016	Thailand	V. parahaemolyticus	AMP, PIP, KF	5,252,400	45.2	45	53	994,428	258,124	4,821	SAMN28767362	GCA_024745395	JANFSK000000000	SRR19513213
ISF-221-5	2016	Cuba	V. parahaemolyticus	AMP, KF, TE, OT (PIP, CTX, CEF, E, ENO)	5,352,130	45.3	114	58	2,428,508	390,904	5,042	SAMN28767363	GCA_024745355	JANFSJ000000000	SRR19513212
ISF-221-6	2016	Cuba	V. parahaemolyticus	AMP, PIP, KF, TE, OT	5,346,813	45	68	82	1,451,948	248,879	4,995	SAMN28767364	GCA_024745305	JANFSI000000000	SRR19513211
ISF-232-1	2016	Thailand	V. parahaemolyticus	AMP, KF, CTX, CEF (E)	5,109,422	45.3	30	129	636,700	116,289	4,709	SAMN28767365	GCA_024745335	JANFSH000000000	SRR19513210
ISF-234-6	2016	India	V. parahaemolyticus	AMP, TE, OT	5,479,698	45	30	190	625,694	71,806	5,081	SAMN28767366	GCA_024745265	JANFSG000000000	SRR19513253
ISF-235-5	2016	India	V. parahaemolyticus	AMP, TE, OT	5,525,342	45.0	80	138	1,757,450	91,505	5,175	SAMN28767367	GCA_024745255	JANFSF000000000	SRR19513252
ISF-237-6	2016	India	V. cholerae	(IPM, E, OT)	4,078,171	47.5	105	82	1,578,896	348,811	3,810	SAMN28767368	GCA_024745295	JANFSE000000000	SRR19513251
ISF-237-7B	2016	India	V. parahaemolyticus	AMP, KF (CTX, CEF, E, ENO)	5,564,410	45.2	85	89	1,732,466	205,604	5,207	SAMN28767369	GCA_024745215	JANFSD000000000	SRR19513250
ISF-242-5	2017	Thailand	V. parahaemolyticus	AMP, KF, CTX, CEF, K (E)	5,529,472	45.0	93	107	1,894,620	216,134	5,214	SAMN28767370	GCA_024745225	JANFSC000000000	SRR19513249
ISF-242-6	2017	Thailand	V. parahaemolyticus	AMP, KF, CTX, CEF (K, E, OT)	5,245,179	45.2	107	62	2,054,414	306,059	4,870	SAMN28767371	GCA_024745155	JANFSB000000000	SRR19513248
ISF-242-7	2017	Thailand	V. parahaemolyticus	AMP, PIP, KF, CTX, CEF, K (E)	5,811,791	44.8	68	86	1,639,176	205,369	5,442	SAMN28767372	GCA_024745175	JANFSA000000000	SRR19513246
ISF-242-8	2017	Thailand	V. parahaemolyticus	AMP, KF, CTX, CEF, SF,(PIP, S, SXT, E, OX)	5,594,092	45.0	34	148	753,934	106,759	5,268	SAMN28767373	GCA_024745165	JANFRZ000000000	SRR19513245
ISF-242-9	2017	Thailand	V. parahaemolyticus	AMP, KF, CTX, CEF (K, E)	5,246,136	45.2	151	50	2,943,230	331,149	4,872	SAMN28767374	GCA_024745115	JANFRY000000000	SRR19513244
ISF-242-10B	2017	Thailand	V. parahaemolyticus	AMP, PIP, KF, CTX, CEF (E, ENO)	5,214,000	45.3	27	111	596,650	109,782	4,820	SAMN28767375	GCA_024745135	JANFRX000000000	SRR19513243
ISF-243-9	2017	India	V. cholerae	(IPM)	4,041,506	47.5	97	101	1,441,966	254,697	3,763	SAMN28767376	GCA_024745065	JANFRW000000000	SRR19513242
ISF-256-8	2017	Thailand	V. cholerae	None	3,907,993	47.6	188	76	2,819,586	247,517	3,635	SAMN28767377	GCA_024745095	JANFRV000000000	SRR19513241
ISF-270-13	2017	Ecuador	V. parahaemolyticus	AMP, TE, OT	5,192,289	45.2	34	135	923,272	119,550	4,826	SAMN28767378	GCA_024745055	JANFRU000000000	SRR19513240
ISF-271-2	2017	India	V. cholerae	None	4,169,508	47.5	73	132	1,103,922	216,279	3,938	SAMN28767379	GCA_024744955	JANFRT000000000	SRR19513239
ISF-318-1	2018	Ecuador	V. parahaemolyticus	AMP, KF, TE, OT (CEF, E)	5,091,952	45.2	53	62	979,238	288,964	4,723	SAMN28767380	GCA_024744985	JANFRS000000000	SRR19513238
ISF-328-1	2019	Mexico	V. diabolicus	AMP, S, SXT, SF, TE, OT (ENO, OX)	5,139,724	44.9	79	72	1,434,814	130,541	4,708	SAMN28767381	GCA_024744965	JANFRR000000000	SRR19513237
ISF-331-1	2019	Bangladesh	V. parahaemolyticus	None	5,398,934	45.4	62	183	1,206,868	61,297	5,067	SAMN28767382	GCA_024744975	JANFRQ000000000	SRR19513235
ISF-332-2	2019	Vietnam	V. parahaemolyticus	AMP, SXT, SF, OT (TE)	5,088,365	45.4	54	93	998,334	128,214	4,750	SAMN28767383	GCA_024745005	JANFRP000000000	SRR19513234

a AMP, ampicillin; CEF, ceftiofur; CTX, cefotaxime; C, chloramphenicol; CIP, ciprofloxacin; DOR, doripenem; ETP, ertapenem; E, erythromycin; ENO, enrofloxacin; GN, gentamicin; IPM, imipenem; K, kanamycin; KF, cephalothin; MEM, meropenem; NOR, norfloxacin; OT, oxytetracycline; OX, oxolinic acid; PIP, piperacillin; PB, polymyxin B; S, streptomycin; SF, sulfisoxazole; SXT, sulfamethoxazole-trimethoprim; TE, tetracycline. Brackets, intermediate resistance; none, no resistance.

For sequencing, stocks were streaked onto TSA-2N and incubated overnight at 35°C; a single colony was used to inoculate 5 mL tryptic soy broth with 2% NaCl (TSB-2N) at 35°C for 24 h, and DNA was extracted using the Maxwell 16 SEV cell DNA purification kit (Promega, Madison, WI, USA). Paired-end Illumina whole-genome sequencing (WGS) was performed using indexed Nextera XT libraries run on a MiSeq instrument (2 × 300 cycles, v3 chemistry) according to the manufacturer (Illumina, San Diego, CA, USA). Reads were trimmed to remove adapters, quality filtered, and error corrected (BBMap v38.26 [BBDuk and Tadpole]) (https://sourceforge.net/projects/bbmap) for *de novo* assembly (SKESA v2.3, Pilon v1.22) (9), gene prediction (Prodigal [commit fe80417]) (10), and metrics reporting (QUAST v5.0.0) (11). Mash (v2.2; Mash Screen) (12) was used for taxonomic assignment of assemblies based on the highest RefSeq (v93; https://www.ncbi.nlm.nih.gov/refseq) identities >0.8 (excluding hits containing the words “phage” or “plasmid”) with V. diabolicus designated according to reference 13 and www.ncbi.nlm.nih.gov/genome. Analysis tools were used with default settings unless noted.

Isolate and genome details are listed in Table 1. In alignment with reference 14, the *de novo* genome assemblies reported here comprised 29 to 238 contigs and coverage depths of 25-fold to 223-fold. Total genome size ranged from 3.9 Mbp to 6.0 Mbp, containing 3,442 to 5,391 predicted genes. The 92 genomes were analyzed and identified as V. parahaemolyticus (*n* = 59), V. diabolicus (*n* = 12), V. cholerae (*n* = 10), V. alginolyticus (*n* = 7), *V. campbellii* (*n* = 1), V. harveyi (*n* = 1), V. owensii (*n = *1), and V. vulnificus (*n* = 1).

### Data availability.

Data for this study have been deposited in NCBI under BioProject accession number PRJNA843723; raw reads and genome accession numbers are listed in Table 1.
